# Predicting progression-free survival in glioblastoma with neuroimaging and machine learning

**DOI:** 10.1007/s11060-026-05650-z

**Published:** 2026-05-28

**Authors:** Davin A. Hickman-Chow, Patrick H. Luckett, Michael Olufawo, Donna Dierker, Joshua S. Shimony, Eric C. Leuthardt

**Affiliations:** 1 Department of Neurological Surgery, Washington University School of Medicine, St. Louis, MO 63110 USA; 2 Mallinckrodt Institute of Radiology, Washington University School of Medicine, St. Louis, MO USA; 3 Brain Tumor Center at Siteman Cancer Center, Washington University School of Medicine, St. Louis, MO USA; 4 Department of Biomedical Engineering, Washington University in Saint Louis, St. Louis, MO 63130 USA; 5 Department of Mechanical Engineering and Materials Science, Washington University in Saint Louis, St. Louis, MO 63130 USA; 6 Center for Innovation in Neuroscience and Technology, Washington University School of Medicine, St. Louis, MO 63110 USA; 7 Brain Laser Center, Washington University School of Medicine, St. Louis, MO 63110 USA; 8 National Center for Adaptive Neurotechnologies, 660 South Euclid Avenue Campus Box 8057, St. Louis, MO 63110 USA

**Keywords:** Deep learning, Glioblastoma, Functional MRI, Progression-Free Survival, Functional Connectivity

## Abstract

**Purpose:**

Glioblastoma (GBM) is the most prevalent and aggressive form of malignant glioma. Reliable estimation of progression-free survival (PFS) prior to medical intervention could strengthen clinical decision-making and improve patient care. Here, we utilize machine learning (ML) to predict PFS in GBM patients using resting state network (RSN) connectivity before medical intervention.

**Methods:**

GBM patients (*N* = 45, mean age 62.1 ± 10.3 years, mean PFS 9.5 ± 5.6 months, 62.2% male) were retrospectively recruited from Washington University Medical Center. All patients completed structural neuroimaging and resting-state functional MRI before surgery. Deep neural networks were trained on resting-state functional connectivity to predict PFS. Feature selection identified the 15 strongest predictive features prior to training.

**Results:**

Sex (*p* = 0.0037), overall survival (*p* = 0.0003), MGMT promoter methylation status (*p* = 0.0064), presentation of weakness (*p* = 0.0037), and presentation of memory impairment (*p* = 0.045) were significantly associated with PFS. Tumor frequency and spatial correlation analyses associated dorsal attention, visual, frontal-parietal, and default mode networks with shorter PFS. Conversely, right-temporal lobe tumors were associated with better outcomes. RSN spatial maps revealed widespread alterations in association networks in GBM patients relative to controls. MRMR feature selection identified thalamic and association network connectivity, including somatomotor, ventral and dorsal attention, and default mode/parietal memory as the strongest predictors of PFS. Using leave-one-out validation, the model predicted PFS with an RMSE of 1.26 months, MAE of 1.08 months, and R² of 0.96 (*p* < 0.001).

**Conclusions:**

Our findings indicate that GBM alters functional brain organization on a widespread scale, and these global effects are informative of patient outcomes.

**Supplementary Information:**

The online version contains supplementary material available at 10.1007/s11060-026-05650-z.

## Introduction

Glioblastoma (GBM) is a primary central nervous system malignancy accounting for approximately 80% of all primary malignant brain tumors in adults, with a reported one-year overall survival around 68% with the highest incidence in patients ≥ 65 years [1]. Despite ongoing efforts to produce novel therapeutic approaches, survival improvements remain minimal, alongside variability in treatment response hindering clinicians’ ability to estimate individual patient outcomes. Limitations on therapeutic efficacy contribute to uncertainty among patients and providers about expected disease course and prognosis following diagnosis. Progression-free survival (PFS), the interval between the first post-radiotherapy MRI scan as the baseline and the date of disease recurrence or death (median ~ 7 months), is widely used in clinical trials as a measure of treatment efficacy [1–3]. Given the short survival window, methods capable of predicting PFS *prior* to surgical intervention could improve prognostic accuracy and inform clinical decision-making.

Preoperative MRI is essential for surgical planning, where optimal outcomes require balancing extent of resection with neurological function and quality of life [4–6]. Multiple MRI-based approaches have been utilized for prognostic assessment in GBM, including radiomics, diffusion MRI, and structural MRI [7, 8]. Structural MRI provides information about tumor size and location, and structural features such as cortical thickness have also been associated with disease outcomes across multiple neurological and psychiatric conditions, including high-grade glioma [2, 9–11]. However, structural measures alone do not capture network-level alterations. Within the expanding literature of MRI modalities aimed at predicting PFS, functional MRI offers network level perspectives by capturing functional disruptions beyond focal structural tumor boundaries [12]. Furthermore, blood oxygen level-dependent (BOLD) resting-state fMRI (RS-fMRI) can represent specific cognitive functions and characterize functional alterations associated with healthy aging and neurodegenerative diseases [13–16]. In brain tumor patients, RS-fMRI has demonstrated potential for presurgical functional network delineation and as a prognostic biomarker [17–20].

Survival modeling in GBM has historically relied on regression-based survival and hazard models. Although these methods have provided valuable insights into survival patterns and associated risk factors, modern ML approaches can handle highly dimensional, complex data and model nonlinear interactions without requiring assumptions about data characteristics [17, 21]. ML has been extensively applied in neuro-oncology, from MRI-based tissue segmentation to radiomic analyses that predict clinically relevant features such as tumor genetics and transcriptomic subtypes [13, 22–25]. Given the predictive capability of ML with the biological and clinical information embedded in functional network organization, we hypothesize that models trained on functional MRI data can provide prognostic information relevant to PFS.

In this study, we apply ML to RS-fMRI to predict PFS at initial diagnosis in 45 patients with GBM. We trained deep feed-forward neural networks using only preoperative FC features derived from 15 resting-state networks (RSNs). This approach has the potential to inform surgical planning, guide personalized therapy strategies, support shared decision-making, and enhance clinic trial design for GBM patients.

## Methods

### Patients

Patients with GBM (*N* = 45) were retrospectively recruited from the Washington University School of Medicine neurosurgery brain tumor service. All cases were histopathologically confirmed as GBM based on surgical specimens evaluated by the Division of Neuropathology between May 2012 and September 2020. Patients treated with biopsy only (i.e., without resection) were excluded. Inclusion criteria included the first occurrence of a brain tumor, biopsy or surgical treatment, and the availability of pre-surgical structural and functional MRI. Exclusion criteria included patients younger than age 18 and patients lost to follow-up. Diagnosis was achieved based on the presence of histomorphological and immunohistochemical characteristics supportive of GBM using the appropriate WHO 2007 and 2016 guidelines [26, 27], including the presence of tumor cells with astrocytic-like appearance, microvascular proliferation, palisading necrosis, pleomorphic hyperchromatic nuclei, and frequent mitoses. All patients in this cohort had a documented radiographic progression and recurrence date with no censored PFS observations. The data contained no IDH1 mutants. The Washington University in St. Louis Institutional Review Board approved this study and waived informed consent due to the retrospective design.

### Clinical characteristics

Demographics and clinical characteristics can be summarized in Online Resource 1. Variables included age and sex, alongside clinical characteristics including PFS (time between the first post-radiotherapy MRI scan and recurrence [3]), overall survival (time between the first post-radiotherapy MRI scan and death), extent of resection (EOR) recorded as gross total resection (GTR), subtotal resection (STR), near total resection (NTR), and laser interstitial thermal therapy (LITT), tumor location, Karnofsky Performance Status (KPS) [20], and tumor genetics (MGMT [28], EGFR [29], TERT [30], IDH1 [31], and PTEN [32]). Medical comorbidities (Hx), including but not limited to alcohol use disorder, tobacco use, hypertension, hyperlipidemia, chronic kidney disease (CKD), cardiac disease (myocardial infarction, arrhythmia, valvular dysfunction), deep vein thrombosis or pulmonary embolism (DVT/PE), psychiatric disorders (Psych), visual deficits, stroke, obesity (BMI > 30), diabetes, and presentation symptoms (Pw) of each patient, including weakness, visual changes, aphasia, confusion, memory impairment, and seizures, were obtained (Online Resource 1). Treatment regimens included patients treated with the Stupp protocol [33] and radiotherapy. Genetic data was measured by the Foundation Medicine commercial laboratory [34] and the Washington University Genomics and Pathology service. Demographics and clinical characteristics are intended to be descriptive and hypothesis-generating.

### Defining tumor recurrence

To determine PFS tumor recurrence was defined using the updated RANO 2.0 criteria for high-grade gliomas [3]. For patients with a post-radiotherapy (post-RT) baseline, response was assessed by comparing follow-up imaging to the post-baseline scans to evaluate for tumor progression or stability. Measurable lesions were defined as contrast-enhancing lesions with clearly defined margins, visible on two or more axial slices, with perpendicular measurements greater than 10 mm. For these lesions, progression was defined as a 25% or more increase in the sum of perpendicular products of enhancing lesions, even in the setting of stable or increasing steroid doses. Furthermore, non-enhancing FLAIR/T2W lesions were evaluated; a significant increase in these lesions, attributable to tumor pathology rather than comorbid factors, also qualified as progression. Clinically, PFS was calculated as the interval between the first post-radiotherapy MRI scan and the date of recurrence or death, with recurrence further validated by clinical deterioration not attributed to steroid decreases [3]. MRI acquisition and processing is further described in Online Resource 2.

### Functional connectivity

Regions of interest (ROI) were used to generate similarity maps for 15 RSN based on previously published results [35]. ROIs were not excluded based on tumor proximity. ROIs were developed by taking each network’s top voxelwise probabilities (> 0.8). RSNs include dorsal somatomotor (SMD), inferior somatomotor (SMI), cinguloopercular (CON), auditory (AUD), default mode (DMN), parietal memory (PMN), visual (VIS), frontoparietal (FPN), salience (SAL), ventral attention (VAN), dorsal attention (DAN), medial temporal (MTL), reward (REW), thalamus (THA), and basal ganglia (BGA). The similarity between networks was calculated by computing the Pearson correlation between the network-specific ROIs, resulting in 120 within and between-network similarity measures. For each subject, voxelwise time series were extracted within every network-specific ROI. Between-network similarity for a network pair (i, j) was computed as the mean of the voxel-to-voxel Pearson correlation matrix formed between the time-series sets from networks i and j (pairwise correlations computed over time, averaged across all voxel pairs). Within-network similarity was computed as the mean of the lower triangle of the correlation matrix formed from all voxels in the network. Prior to correlation, missing or non-finite time-series values were imputed with the ROI-wise mean.

### Machine learning and statistical analysis

Analyses were performed in either MATLAB R2021b or R 4.2.1. Minimum Redundancy Maximum Relevance [36] (MRMR) feature selection was used to identify the most informative and non-redundant subset of FC features for model training. MRMR ranks features by jointly maximizing their correlation with the outcome variable (maximum relevance) while minimizing inter-feature redundancy, thereby promoting selection of complementary predictors. Given the relatively small cohort size, feature selection was performed prior to model training to avoid overfitting and improve generalizability. From the complete set of FC features, only the top 15 features (corresponding to 80% variance explained by PCA) identified by MRMR were used as inputs to the models and were selected as a dimensionality-control to retain substantial information from the FC feature space while keeping the predictor count modest relative to the cohort size. This threshold was chosen to balance information retention against overfitting risk, rather than as an unrestricted performance-optimization step. MRMR feature selection was also performed independently within each LOOCV training fold. For each iteration, the held-out subject was excluded before feature ranking was conducted, and MRMR was applied using only the training subjects in that fold. The selected features were then used to train the model and predict the held-out subject. The top 15 predictors were found to be consistent between each fold resulting in marginal variability between the folds. Further, an average per-network feature weight was generated by averaging all within and between-network feature weights for each given network (e.g., to calculate the average feature weight for SMD, we averaged all feature weights associated with SMD [SMDxSMD, SMDxSMI, SMDxCON….]). Lastly, voxelwise FC feature maps were calculated by taking the dot product of the average feature weights with publicly available FC probability maps [35].

PFS prediction was achieved using deep feedforward artificial neural networks (ANN). The network architecture consisted of an input layer followed by three fully connected layers, each separated by a nonlinear activation function (sigmoid) and layer normalization. All models were trained using a quasi-Newton L-BFGS optimizer. To identify optimal model configurations, a systematic hyperparameter search was performed. First, models were initialized with random weights to mitigate the influence of local minima. Second, networks with varying hidden layer sizes (5, 10, or 15 neurons per layer) were evaluated. Third, alternative loss functions were tested, including mean squared error (MSE), mean absolute error (MAE), and Huber loss. For each configuration, training was repeated approximately 50 times. Model performance was evaluated based on both training and validation accuracy. The best-performing model within a configuration was retained; whenever a new model yielded superior performance, it replaced the prior best model. This iterative optimization procedure was repeated until convergence on the most accurate model architecture.

Models were trained using a leave-one-out-cross-validation (LOOCV) framework, ensuring unbiased evaluation of each subject. Data augmentation was applied exclusively within the training set to prevent leakage into the held-out sample. Augmentation consisted of two randomization strategies: (1) selecting 70–80% of available time points for FC estimation, and (2) selecting 70–80% of voxelwise ROIs from which the final FC averages were computed. For each optimization cycle described above, a random number of augmented training samples (ranging from 10 to 500) was generated, such that each newly initialized model was trained on a distinct set of augmented input-output pairs. During this process, a randomly sampled subset of the augmented training data (5–20%) was reserved for validation termination, ensuring no overlap with the held-out LOOCV subject. PFS was reported in months, and outcome outliers (*n* = 2) were clipped at 24 months.

**Fig. 1:**
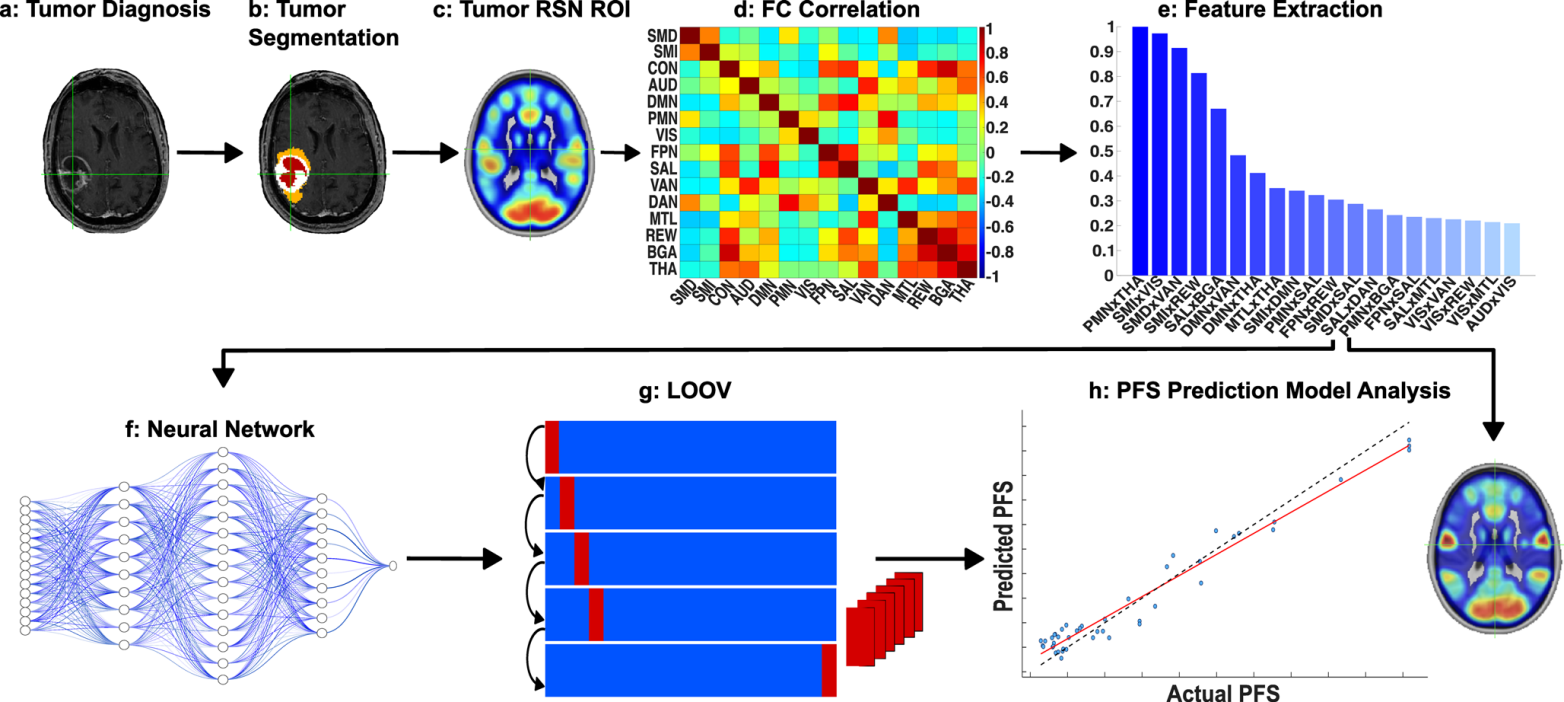
Progression-free survival prediction pipeline. Structural and resting-state MRI are collected at the time of diagnosis and processed for data analysis (**a**). Tumors are then segmented (**b**), and RSNs are separated (**c**), correlated (**d**), and fed into an MRMR algorithm for feature extraction (**e**). Selected features are used to train a deep feed-forward neural network (**f-g**) under LOOCV. Models are then analysed for PFS prediction accuracy and brain mapping (**h**)

To quantify network-level differences between GBM patients and healthy individuals, we computed effect sizes for each RSN using previously published RSN probability maps derived from a large cohort of 2,000 controls processed with a 3D convolutional neural network [35]. Tumor patients underwent the same 3D-CNN mapping procedure, and their RSN probability maps were averaged across subjects to generate a comparable group-level representation. For each RSN, we then calculated Cliff’s delta between the control and tumor mean probability distributions. This approach assesses the degree of non-overlap between the RSN probability distributions of the two groups and provides a standardized measure of how strongly a given network’s spatial organization differs in GBM. Because this analysis is based on group-averaged RSN probability maps rather than individual-level values, the resulting effect sizes reflect the magnitude of network-wide alterations in overall RSN expression rather than subject-specific variability. Effect sizes were interpreted as: |d| < 0.33 = negligible/small, 0.33–0.47 = moderate, and ≥ 0.47 = large. The PFS prediction pipeline and methods described above can be found in Fig. 1.

## Results

Online Resource 1 provides information regarding cohort demographics, Karnofsky Performance Scale (KPS), tumor location, molecular features, patient medical history, and presentation of symptoms at the time of diagnosis. EOR was not significantly associated with PFS (Online Resource 1). The cohort was predominantly male (62.2%), with mean age at diagnosis 62.1 ± 10.3 years. Mean and median PFS were 9.5 and 6.8 months, respectively. Of the measured variables, sex (*p* = 0.0037), overall survival (*p* = 0.0003), MGMT promoter methylation status (*p* = 0.0064), presentation of weakness (*p* = 0.0037), and presentation of memory impairment (*p* = 0.045) were associated with PFS. Online Resource 3 shows cohort-level tumor frequency maps calculated by averaging the tumor segmentation maps, as well as the mean frequency per RSN. Tumors most frequently involved BGA, CON, and FPN.

**Fig. 2:**
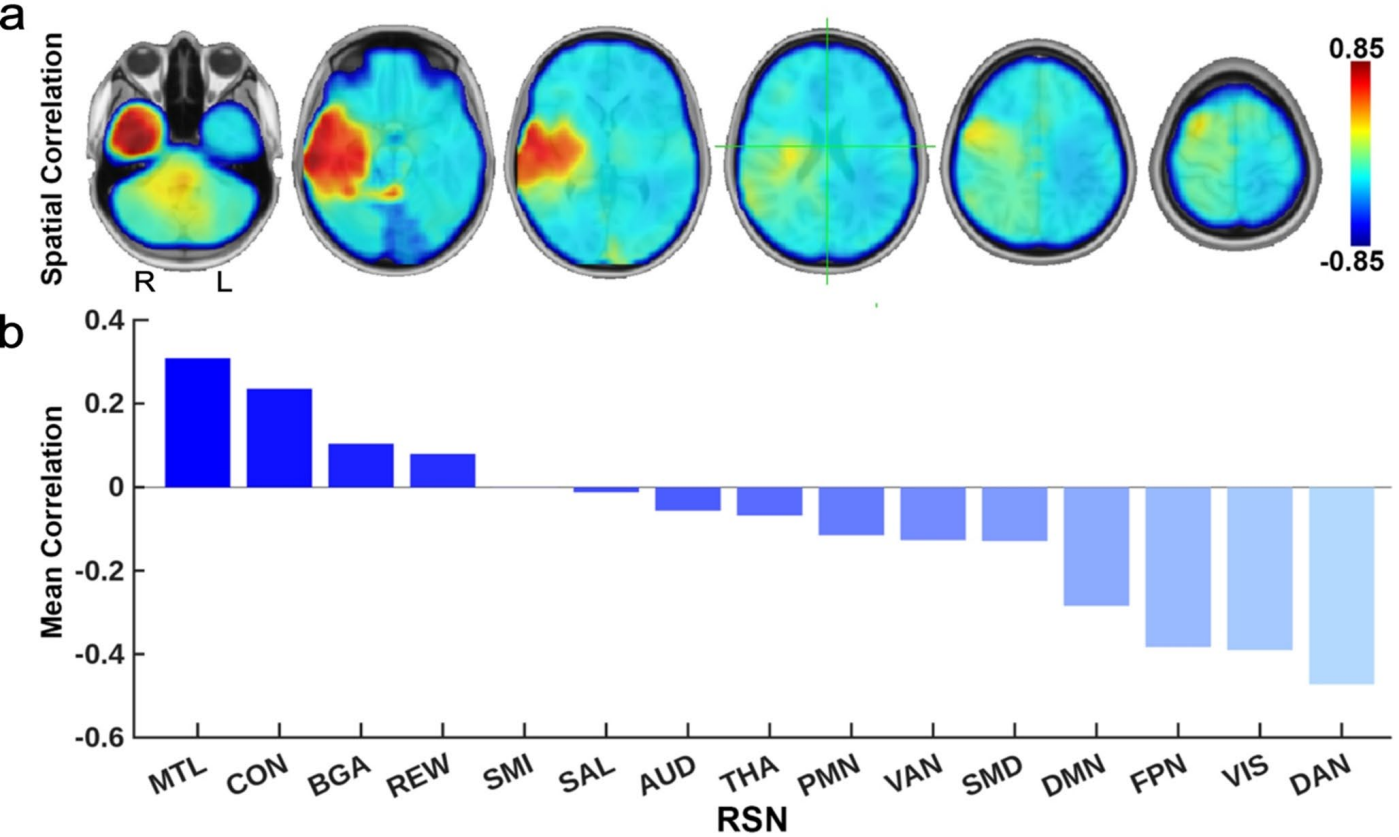
Spatial association of PFS with tumor frequency (**a**) Voxelwise spatial pattern of association between subject-level PFS and tumor segmentation maps. Red indicates regions associated with longer PFS. (**b**) Correlations averaged per RSN

Figure 2 shows the spatial and ROI correlations of the tumor segmentation maps with PFS. Longer PFS was associated with tumors in the right temporal region, and primarily affected MTL, CON, BGA, and REW. Conversely, tumors involving DAN, VIS, FPN, and DMN were associated with shorter PFS. In general, right-hemisphere tumors were associated with longer PFS compared to left-hemisphere tumors.

**Fig. 3:**
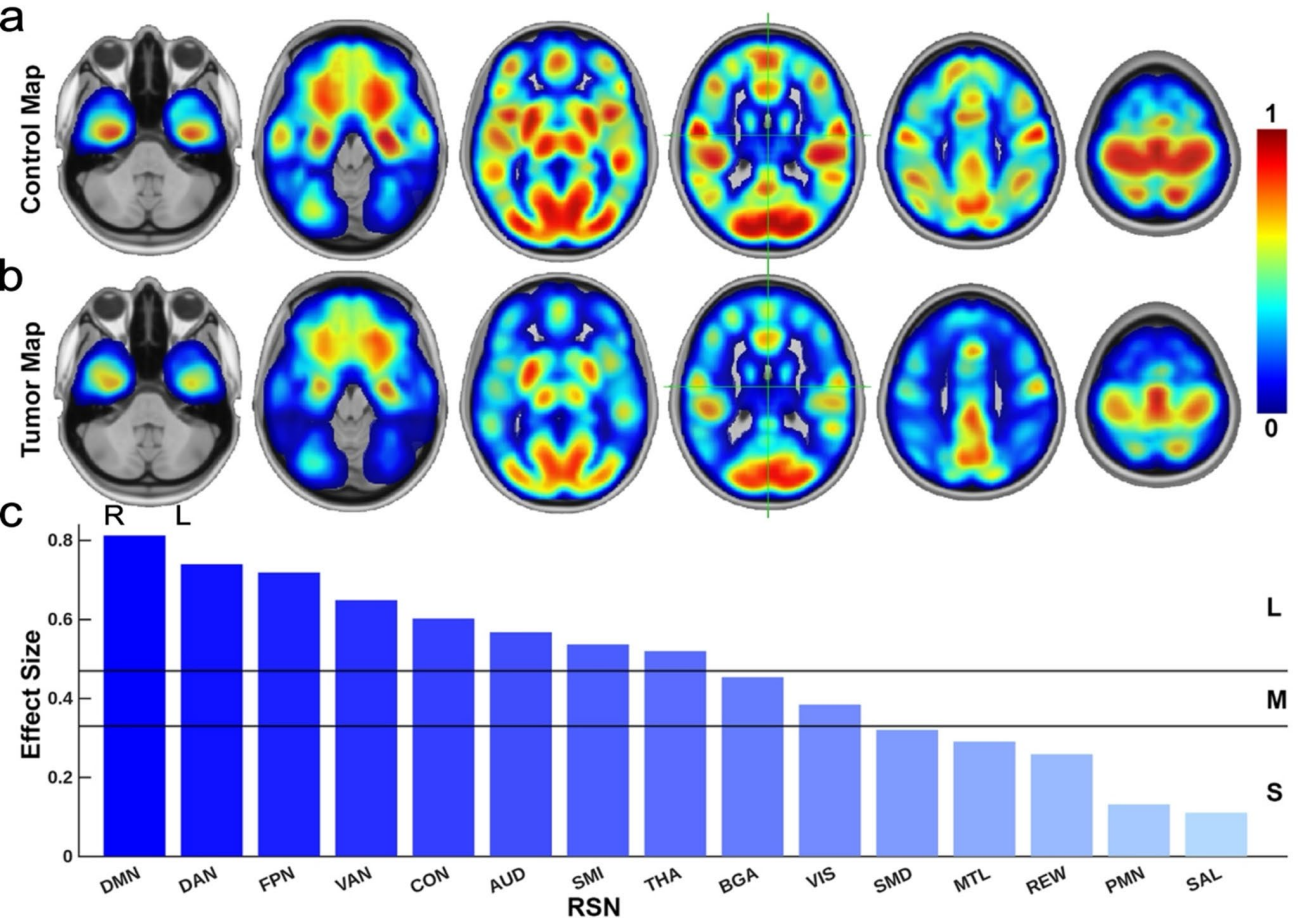
Effect size: controls/acute. (**a**) RSN distribution, averaged across 2,000 control subjects. (**b**) Corresponding RSN distribution averaged across tumor patients. (**c**) Effect size of network-specific probability differences between controls and tumor patients, where positive values indicate higher probabilities and reflect a more normal or healthy network organization

Figure 3 summarizes differences between mean RSN probability maps in healthy controls and GBM patients. Figure 3a shows whole-brain RSN probability maps for controls acquired from previously published work [37], generated by extracting the softmax probability of each voxel’s argmax-assigned network. Larger values represent regions strongly associated with their assigned RSN. Figure 3b presents the corresponding mean maps for tumor patients, and Fig. 3c shows the per-RSN effect size between controls and tumor patients. Cliff’s delta effect sizes [38] indicated the largest group differences occurred in DMN (0.81), DAN (0.74), FPN (0.72), VAN (0.65), CON (0.60), AUD (0.57), SMI (0.54), and THA (0.52). All networks showed reduced probabilities in tumor patients relative to controls, indicating global disease burden.

**Fig. 4:**
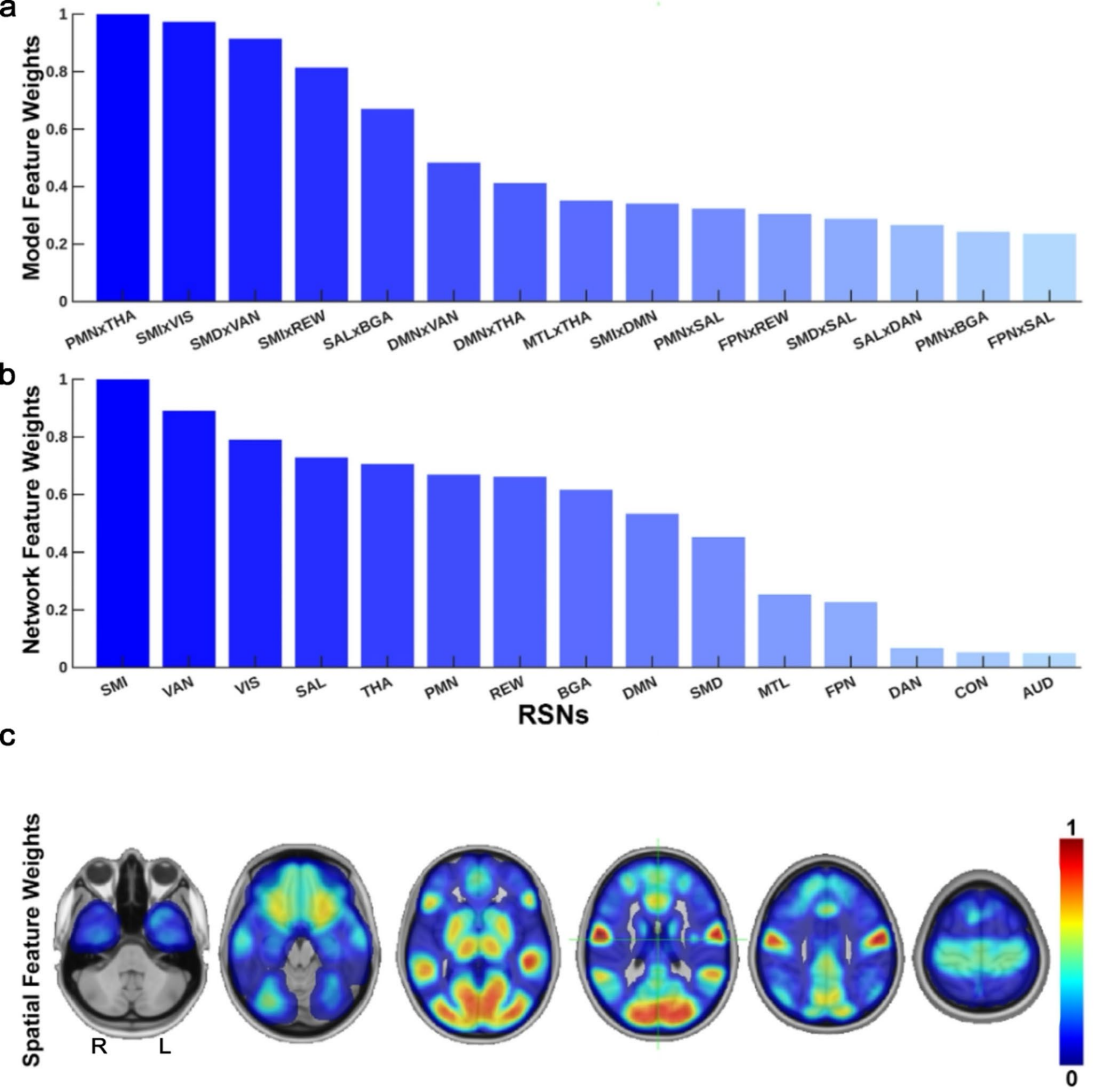
MRMR feature selection. (**a**) The top 15 network correlation features/weights identified by MRMR and used for model training. (**b**) Network-level feature weights computed by averaging weights of features involving each RSN. (**c**) Network-level features mapped onto spatial RSN distributions

Figure 4a shows the MRMR feature selection results. The top 15 RSN features used for model training included PMNxTHA, SMIxVIS, SMDxVAN, SMIxREW, SALxBGA, DMNxVAN, DMNxTHA, MTLxTHA, SMIxDMN, PMNxSAL, FPNxREW, SMDxSAL, SALxDAN, PMNxBGA, and FPNxSAL. Figure 4b shows the feature weights (all features) averaged on a per-RSN basis, with the top networks including SMI, VAN, and VIS. Figure 4c shows the results of mapping those feature weights onto the spatial RSN distributions.

**Fig. 5:**
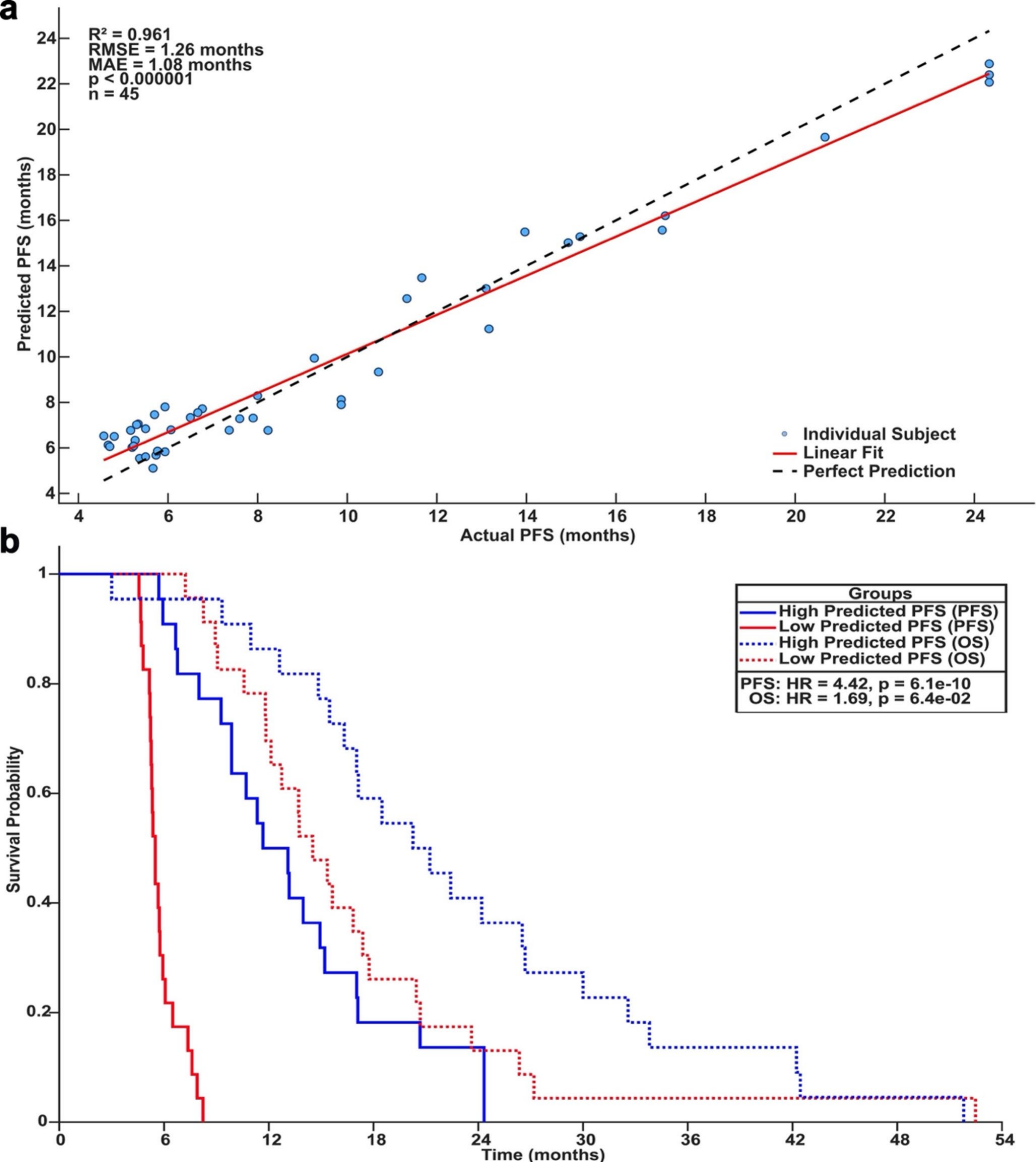
PFS prediction results. (**a**) Actual vs. predicted PFS. (**b**) Kaplan-Meier progression-free (PFS) and overall survival (OS) analysis

Lastly, Fig. 5 shows the LOOCV performance for PFS prediction. Across the LOOCV models used to generate the primary results, 30 models used 5 neurons per hidden layer, while the remaining 15 used 10 neurons per hidden layer. Overall, despite the small sample size, the model achieved mean absolute error of 1.08 months and an R^2^ of 0.96 (Fig. 5a). Supplemental Fig. 2 (Online Resource 4) shows distributions of prediction results. Furthermore, an additional linear model, using the same MRMR-selected features and identical LOOCV framework, was used for evaluation and comparison of the ANN. In comparison, the trained linear model showed worse predictive performance (MAE = 8.93 months), supporting the use of deep ANNs. These findings indicate the model was able to accurately predict PFS using only 15 RSN features and no demographic/genetic information. Figure 5b shows Kaplan-Meier analysis with subjects stratified into high (*n* = 22) and low (*n* = 23) predicted PFS groups using the median predicted value (7.3 months) as a cutoff. For PFS, patients classified into the low predicted group had a 4.42-fold increased hazard of progression (p-value < 0.001), while overall survival showed a smaller 1.69-fold (*p* = 0.064) increased hazard of progression. This further demonstrates the model’s efficacy in prediction and stratification of PFS.

## Discussion

In this study, we used preoperative RS-fMRI and ML to predict PFS in GBM patients. RSN-based FC features enabled accurate PFS estimation without demographic or clinical variables, while tumor frequency and RSN probability analyses linked network disruption and tumor location to PFS. These findings support GBM as a network-level disease and highlight preoperative FC as a promising noninvasive biomarker for individualized risk stratification and clinical decision-making.

Our findings are consistent with prior work showing GBM disrupts multiple RSNs [2, 39, 40]. RSN probability comparisons between patients and controls (Fig. 3) revealed reductions across sensorimotor, attentional, and higher-order association systems, with the largest differences in DMN, DAN, FPN, VAN, and CON ࣧ large-scale hubs supporting cognition and executive control [39]. These disruptions likely reflect tumor-mediated edema, white matter tract damage, and diaschisis, producing functional changes that extend beyond focal lesion boundaries [40]. The pervasive nature of these disruptions highlights that GBM impacts the brain at system levels.

While resting-state network connectivity has been shown to predict overall survival [41], predicting PFS is more specific to the biologic response to therapy. Overall survival integrates tumor biology with host factors, comorbidities, and tolerance to treatment. Thus, that FC carries prognostic information about when the tumor will grow back after surgery and chemoradiation, and that this prognostic information extends beyond what can be inferred from tumor location and demographics alone, speaks to the brain-wide nature of GBM as a disease. Tumor frequency maps (Online Resource 3) demonstrated involvement of subcortical and association networks, including BGA, CON, and FPN. DAN, VIS, FPN, and DMN networks was associated with shorter PFS, while right temporal lobe involvement correlated with longer PFS, consistent with previous literature [42, 43]. Conversely, FC-derived features provide a complementary, global measure of network integrity capturing distributed effects not evident on conventional structural imaging [43, 44]. Together, these results reinforce GBM as a systems-level disease in which functional disorganization, rather than focal involvement alone, is a fundamental aspect of the cancer’s oncologic behavior, making FC a promising biomarker for capturing the diffuse network vulnerability underlying variable tumor recurrence timelines.

MRMR features offer additional insight into the network vulnerabilities that shape clinical outcomes in GBM. Many top-ranked predictors (Fig. 4) involved interactions between high-level association networks, such as DMN, VAN, FPN, and SAL, and subcortical structures, including the thalamus and basal ganglia. This suggests that prognosis is influenced by disruption within individual networks and by the integrity of cross-network communication particularly between cortical hubs and subcortical relay systems essential for large-scale coordination [45]. The prominence of somatomotor, visual, and ventral attention contributions in the network-level feature weights (Fig. 4b) suggests that both primary and association systems may be susceptible to GBM-related disruptions. Collectively, these feature patterns are biologically interpretable within existing models of GBM as a disorder of distributed network dysfunction [46, 47], while highlighting specific networks and connections whose disruption may describe the biological extent to which the tumor is invading the brain and contribute disproportionately to differences in patient outcomes.

The ability to estimate PFS from preoperative imaging alone has direct implications for surgical planning and patient counseling. Prognosis influences resection aggressiveness near the eloquent cortex and anticipated recovery. A noninvasive biomarker reflecting underlying network integrity could help identify patients likely to benefit from extensive intervention versus those for whom a conservative approach is more appropriate. Preoperative PFS estimates can also support informed discussions with patients and families, guide treatment expectations, and stratify candidates for clinical trials. By providing individualized prognostic insight at diagnosis, connectivity-based predictions have the potential to enhance decision-making and quality of care for patients with GBM.

This study is limited by its modest cohort size, which reduced statistical power and restricted subgroup and sensitivity analyses. The 15-feature MRMR approach was chosen to balance information retention with dimensionality control, but future work should test alternative feature counts and machine-learning models. Because demographic, clinical, and molecular variables were excluded to isolate the prognostic value of resting-state FC, larger studies should compare FC-only, clinical-only, and combined multimodal models to determine whether FC provides independent or complementary prognostic value. The observed sex association should be interpreted cautiously and validated in larger cohorts that permit adjustment for demographic and clinical covariates. In addition, one patient underwent LITT, which is not directly comparable to conventional EOR categories. This case was retained in the cohort but was not interpreted as equivalent to a conventional EOR category; accordingly, descriptive treatment-related comparisons should be considered exploratory. Finally, because PFS values were clipped at 24 months to limit the influence of two extreme long-PFS observations in this small cohort, model predictions should be interpreted within the bounded PFS range used for training.

## Conclusion

In summary, this study demonstrates that preoperative resting-state FC has the potential to serve as a noninvasive biomarker of PFS. GBM patients demonstrated widespread reductions in RSN probability organization relative to controls. Our approach enabled reliable prediction of PFS from only 15 connectivity features, without reliance on demographic, genetic, or treatment variables. Importantly, the ability to estimate PFS from preoperative imaging alone provides clinically meaningful information that can support surgical decision-making, risk stratification, and patient counseling.

## Supplementary Information

Below is the link to the electronic supplementary material.

Supplementary Material 1

Supplementary Material 2

Supplementary Material 3

Supplementary Material 4

## Data Availability

The data used in this study will be made available after approval from the appropriate study PIs (Eric Leuthardt, Joshua Shimony).
