# Supplementary material for: Predicting progression-free survival in glioblastoma with neuroimaging and machine learning
Source: J Neurooncol. 2026 May 28;178(1):29. doi: 10.1007/s11060-026-05650-z (PMC13219129; doi:10.1007/s11060-026-05650-z)
Supplement: Supplementary file 1 — Supplementary Material 1 [file 11060_2026_5650_MOESM1_ESM.pdf]

Predicting Progression-Free Survival in Glioblastoma with Neuroimaging and Machine Learning

Journal of Neuro-Oncology

Davin Hickman-Chow[1*] BS, Patrick H. Luckett[1*] PhD, Michael Olufawo[1] MD, MBA, Donna Dierker[2] MS, Joshua S. Shimony[2] MD, PhD, and Eric C. Leuthardt[1,5-9] MD

Corresponding author: Patrick H. Luckett, Davin A. Hickman-Chow

Email: luckett.patrick@wustl.edu, d.a.hickman-chow@wustl.edu

	All Data	Correlation with PFS (r-value)	P-Value
N	45	—	—
Overall Survival(m) ± STD (Median)	19.9 ± 11.1 17.0	0.514	0.0003***
Progression Free Survival ± STD (Median)	9.5 ± 5.6 6.8	—	—
Age(years) at Diagnosis	62.1 ± 10.3	-0.066	0.67
Sex(Male)	62.2%	0.523	0.0037**
KPS>70	88.9%	-0.165	0.56
EOR (mean) ± STD	1.7 ± 0.97	0.055	0.72
GTR	57.8%	-0.045	0.81
NTR	17.8%	-0.00	0.99
STR	22.2%	-0.023	0.92
LITT	2.2%	—	—
Tumor Location			
Frontal Tumor	44.4%	0.008	0.97
Parietal Tumor	31.1%	0.76	0.69
Temporal Tumor	35.6%	-0.119	0.52
Occipital Tumor	13.3%	-0.2	0.44
Cingulate Tumor	0.0%	—	—
Other Tumor	4.4%	0.09	0.85
Molecular Features			
MGMT	37.8%	0.492	0.0064**
TERT	60.0%	0.101	0.58
PTEN	55.6%	0.144	0.42
EGFR	55.6%	-0.15	0.40
IDH1	0.0%	—	—
Patient Medical History			
Hx Obese	24.4%	-0.083	0.69
Hx Diabetes	17.8%	-0.105	0.66
Hx Alcohol use disorder	2.2%	—	—
Hx Tobacco	20.0%	-0.13	0.56
Hx Hypertension	40.0%	0.21	0.23

Hx Hyperlipidemia	26.7%	0.071	0.73
Hx CKD	2.2%	—	—
Hx Cardiac	13.6%	0.184	0.48
Hx DVT/PE	6.7%	0.063	0.87
Hx Psych	8.9%	0.034	0.82
Hx Visual Deficit	2.2%	—	—
Hx of Stroke	4.4%	0.558	0.195
Hx Seizure	2.2%	—	—
Hx Score ± STD	0.3 ± 0.7	-0.036	0.81
Presentation Symptoms			
Pw Aphasia	51.1%	-0.113	0.52
Pw Weakness	26.7%	0.573	0.0037**
Pw Visual Changes	22.2%	-0.129	0.55
Pw Confusion	31.1%	0.053	0.79
Pw HA	28.9%	-0.058	0.77
Pw Memory Imp	11.1%	-0.56	0.045*
PW Seizure	15.6%	0.026	0.93
PW score ± STD	1.9 ± 1.0	0.001	0.99

Supplemental Table 1: Patient Demographics. Continuous data analyzed with Spearman correlation. Binary data analyzed with Wilcoxon rank-sum test. Statistical significance: *$p < 0.05$, **$p < 0.01$, ***$p<0.001$, Hx = History of, Pw = presented with, STD = standard deviation, EOR = extent of resection, GTR = gross total resection, NTR = near total resection, STR = subtotal resection, LITT = laser interstitial thermal therapy, m = months

The primary predictive analysis treated PFS as an observed continuous outcome measured in months, with exploratory association analysis performed using rank-based methods to better accommodate the modest sample size and the potential non-normal distribution of PFS.
