# Supplementary material for: Predicting progression-free survival in glioblastoma with neuroimaging and machine learning
Source: J Neurooncol. 2026 May 28;178(1):29. doi: 10.1007/s11060-026-05650-z (PMC13219129; doi:10.1007/s11060-026-05650-z)
Supplement: Supplementary file 2 — Supplementary Material 2 [file 11060_2026_5650_MOESM2_ESM.pdf]

Predicting Progression-Free Survival in Glioblastoma with Neuroimaging and Machine Learning

Journal of Neuro-Oncology

Davin A. Hickman-Chow[1*] BS, Patrick H. Luckett[1*] PhD, Michael Olufawo[1] MD, MBA, Donna Dierker[2] MS, Joshua S. Shimony[2] MD, PhD, and Eric C. Leuthardt[1,5-9] MD

Corresponding authors: Patrick H. Luckett, Davin A. Hickman-Chow

Email: luckett.patrick@wustl.edu, d.a.hickman-chow@wustl.edu

1.1 MRI Acquisition

Neuroimaging was performed on a Siemens Trio or Skyra 3T MRI scanner. Structural images included T1-weighted (T1w) magnetization prepared rapid acquisition gradient echo (MPRAGE: TE = 2.53 ms, TR = 1900 ms, TI = 900 ms, 256 × 256 acquisition matrix, 0.976 × 0.976 × 1 mm voxels), fluid-attenuated inversion recovery (FLAIR: 2D, slice thickness 5 mm, gap 1 mm, 256 x 256 matrix 0.9 x 0.9 mm pixel size, TE = 129 ms, TR = 8500 ms, TI = 2440 ms, flip angle 130), and T2-weighted (T2w) fast spin-echo (FSE: TE = 93 ms, TR = 5600 ms, 256 × 256 acquisition matrix, 1.093 × 1.093 × 2 mm voxels). Resting state used BOLD EPI sequencing (3 mm^3 isotropic voxels; TE = 27 ms; TR = 2.2 - 2.9 s; field of view = 256 mm; flip angle = 90, time series mean ± STD = 289 ± 45.6).

1.2 MRI Processing

Preprocessing of structural data was performed with FreeSurfer (http://surfer.nmr.mgh.harvard.edu). Segmentation outputs were visually examined for quality assurance. Specifically, T1w and T2w images were inspected to verify that brain structures were free of motion-related artifacts such as blurring, ringing, striping, and ghosting. Three raters reviewed the segmentation to ensure data quality[1]. Preprocessing of fMRI data followed previously described methods, including compensation for slice-dependent time shifts, elimination of systematic odd-even slice intensities, and rigid body correction of head movement[2, 3]. The data were subsequently resampled and registered to 3 mm^3 atlas space via affine alignment to the T1w structural image. Additional steps included voxel-wise removal of linear trends, temporal low-pass filtering to retain frequencies below 0.1 Hz, regression of nuisance signals, and spatial smoothing using a 6 mm full-width at half-maximum Gaussian kernel. Frame censoring was applied based on DVARS, as previously described[2].

Automated tumor segmentation was performed using a pre-trained convolutional neural network (CNN) applied to post-contrast T1w, T2w, and FLAIR scans[4]. The network classified tumor components into vasogenic edema, necrotic/non-enhancing core, and enhancing core. Segmentation outputs underwent visual quality control, and

inadequate results were excluded. The resulting tumor mask was used to constrain registration of structural and functional images. Segmentation maps were further converted into voxel-wise frequency maps. Voxels outside the segmentation were assigned 0, edema was assigned 1, and non-enhancing/enhancing core regions were assigned 2. Aggregation of these maps across individuals produced heat maps quantifying voxel-level tumor involvement within the atlas. These images were subsequently used to create the tumor frequency maps and were used to show the correlation between the tumor frequency and outcome (Online Resource 3). Segmentation outputs were converted into voxel wise tumor-involvement maps across subjects to visualize spatial trends between tumor involvement and PFS. This analysis was intended as an exploratory visualization of tumor-location and PFS rather than voxel-level analysis.

1. Lamichhane B, Luckett PH, Dierker D, et al (2023) Structural gray matter alterations in glioblastoma and high-grade glioma—A potential biomarker of survival. Neuro-Oncology Advances 5:vdad034. https://doi.org/10.1093/noajnl/vdad034

2. Power JD, Barnes KA, Snyder AZ, et al (2012) Spurious but systematic correlations in functional connectivity MRI networks arise from subject motion. NeuroImage 59:2142–2154. https://doi.org/10.1016/j.neuroimage.2011.10.018

3. Shulman GL, Pope DLW, Astafiev SV, et al (2010) Right Hemisphere Dominance during Spatial Selective Attention and Target Detection Occurs Outside the Dorsal Frontoparietal Network. J Neurosci 30:3640–3651. https://doi.org/10.1523/JNEUROSCI.4085-09.2010

4. Isensee F, Kickingereder P, Wick W, et al (2018) Brain Tumor Segmentation and Radiomics Survival Prediction: Contribution to the BRATS 2017 Challenge. https://doi.org/10.48550/arXiv.1802.10508
