# Supplementary material for: Predicting progression-free survival in glioblastoma with neuroimaging and machine learning
Source: J Neurooncol. 2026 May 28;178(1):29. doi: 10.1007/s11060-026-05650-z (PMC13219129; doi:10.1007/s11060-026-05650-z)
Supplement: Supplementary file 3 — Supplementary Material 3 [file 11060_2026_5650_MOESM3_ESM.pdf]

Predicting Progression-Free Survival in Glioblastoma with Neuroimaging and Machine Learning

Journal of Neuro-Oncology

Davin A. Hickman-Chow[1*] BS, Patrick H. Luckett[1*] PhD, Michael Olufawo[1] MD, MBA, Donna Dierker[2] MS, Joshua S. Shimony[2] MD, PhD, and Eric C. Leuthardt[1,5-9] MD

Corresponding authors: Patrick H. Luckett, Davin A. Hickman-Chow

Email: luckett.patrick@wustl.edu, d.a.hickman-chow@wustl.edu

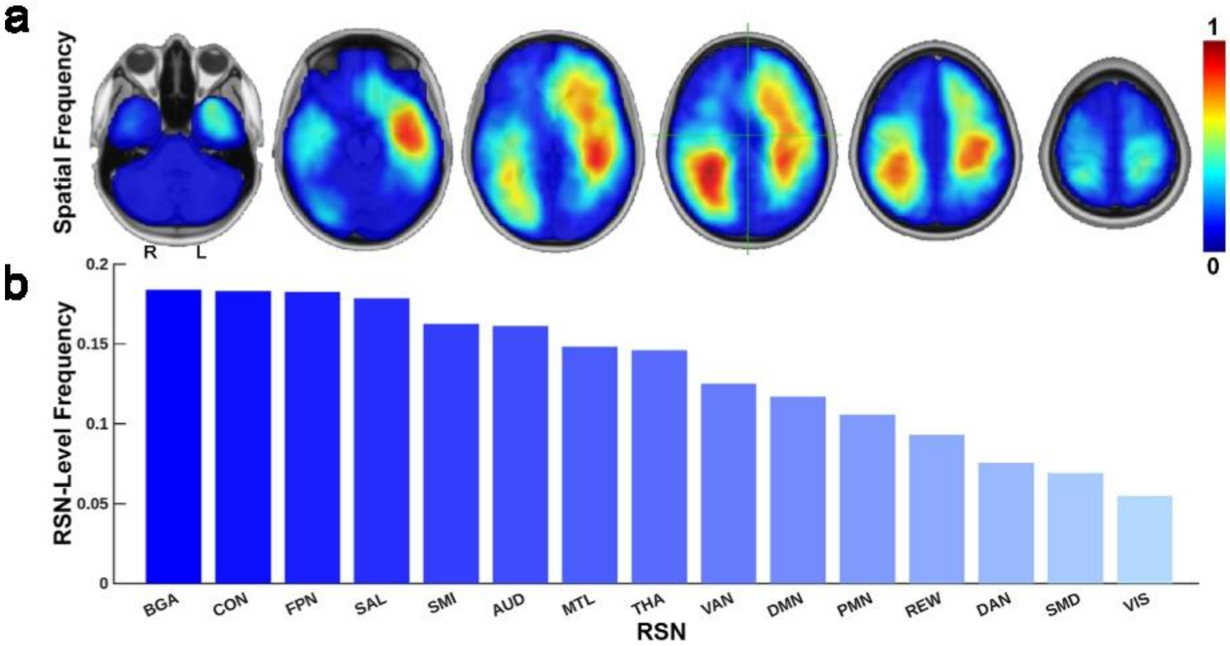

Supplemental Fig. 1 a) Spatial tumor frequency map generated by averaging the tumor segmentation masks. b) Corresponding tumor frequency values averaged within resting-state network (RSN) regions based on the RSN parcellation
